# Liberals and conservatives share information differently on social media

**DOI:** 10.1093/pnasnexus/pgaf206

**Published:** 2025-06-27

**Authors:** Ho-Chun Herbert Chang, James N Druckman, Emilio Ferrara, Robb Willer

**Affiliations:** Program in Quantitative Social Science, Dartmouth College, Hanover, NH 03755, USA; Department of Political Science, University of Rochester, Rochester, NY 14627, USA; Thomas Lord Department of Computer Science, University of Southern California, Los Angeles, CA 90007, USA; Information Sciences Institute, University of Southern California, Los Angeles, CA 90292, USA; Department of Sociology, Stanford University, Stanford, CA 94305, USA

**Keywords:** social media, partisan asymmetries, supply and demand, information diffusion, toxicity

## Abstract

Social media provides citizens with direct access to information shared by politicians. Citizens, in turn, play a critical role in diffusing such content. Do conservative and liberal citizens differ in their decisions about which representatives’ social media content to share? We analyze more than 13 million users’ sharing of 1,293,753 messages by US members of Congress on Twitter from 2009 to 2019, leveraging estimates of users’ political ideology from over 3.5 billion prior retweets. We find that liberals retweeted statements covering a broader range of issues than conservatives. Liberals also shared statements with content rated as relatively more toxic by a standard classifier. Given well-established tendencies toward political homophily among social media users, our results suggest that, compared to conservatives, liberals will be exposed to a more diverse set of issues and more toxic content originating from elected representatives. We conclude with a discussion of several possible explanations for these patterns.

Significance Statement Social media users who engage with politics by following political representatives make decisions about what elite-generated content to share with others. We explore how these decisions are made. We analyze more than a decade of Twitter data, inferring users’ political ideology from their media diet and online behavior. We find evidence that liberals share (measured via retweeting) elite political communications covering more diverse topics (i.e. a wider range of policy topics) than conservatives. Liberals also share more toxic elite-generated content—defined as a rude, disrespectful, or unreasonable comment—than conservatives. These findings suggest that liberals may find themselves in more diverse, but also more toxic, information environments than their conservative counterparts when it comes to social media.

Social media play a crucial role in shaping contemporary political information environments, both in the United States and globally. Audience members influence what political information does or does not become prevalent via their decisions to diffuse social media information to others (1). Do liberals and conservatives make these decisions in similar ways? Sizable literatures study following decisions (e.g. 2, 3), how characteristics of message content (e.g. moral content) affect the likelihood of a message being shared (e.g. 1, 4, 5), and decisions about sharing of misinformation (e.g. 6–10).

We focus on a distinct question about how the (American) audience's ideology conditions decisions about what information, specifically information from elites, to share or diffuse. We define elites as political representatives—members of Congress. This is an important question given that generally (although not entirely) liberals obtain social media information from other liberals and conservatives from other conservatives (2, 11). Thus, if liberals and conservatives who consume elite content pass along distinct types of messages, it could alter downstream information bubbles for others who do not consume elite content. In essence, by choosing to share some elite messages but not others with their networks, individuals shape the broader information ecosystem.

## Ideology and sharing behaviors

The information ecosystem can be seen as the supply and demand of attention, mediated by the underlying platform (i.e. social media). The supply is the amount of content available for users to consume (i.e. posts, videos, stories, ads). The demand for attention refers to the amount of attention that users can allocate to social media content; it is measured by viewership, engagement, and diffusion [12, 13]. In this study, we are specifically interested in capturing diffusion phenomena (e.g. retweeting) rather than endorsement, or whether a retweeter supports a certain stance.

There is reason to expect ideologically divergent diffusion with regard to two information attributes. The first concerns engagement with political issues or policies. Ideologues and partisans tend to care more about some issues relative to others. Specifically, parties and candidates tend to prioritize some “issue bundles” more than others (14, 15), often those on which they are advantaged (16, 17). For instance, it is generally assumed that Democrats tend to prioritize the environment, education, and civil rights, while Republicans put more weight on defense, the economy, and international affairs.

We expect ideologues to react differently to partisan policy agendas. Prior research (18) finds that conservatives tend to prefer situations of reduced uncertainty, threat, and discord. They are generally less open to new experiences, such as being less engaging with new political information in forming evaluations (19), less likely to support change to the status quo in elections (19, 20), and less tolerant of ideological outgroups (21). One could thus infer that conservatives, relative to liberals, would be less open to varying information that counters their standing perspective. Consequently, they would be less likely to share (i.e. retweet) policies that come from the other party's (i.e. liberals’/Democrats’) agenda. The Shy Tory Factor or Shy Elephant Factor describes how electoral votes outperform opinion polls for conservative candidates, which likewise suggests limits to conservative diffusion of information (22). According to Ref. (23), conservatives have less of an interest in sharing issues with which they are not already familiar. We hypothesize that, relative to liberals, when conservatives encounter issues that are not part of their existing agenda in a tweet from a member of Congress (MC ), they will be less likely to retweet it (*Hypothesis 1*).^a^

Hypothesis 1 suggests that Democratic MCs might elicit a less diverse (retweeting) audience than Republican MCs. In contrast, Republican MCs who mostly emphasize conservative issues will draw both a conservative (retweeting) audience and a liberal (retweeting) audience, which is assumed to be more open to variation in agendas (thus, their audience will be more diverse). Thus, a way to consider Hypothesis 1 (and the way we test it) is that Democratic MCs will have less diverse audiences than Republican MCs.

The second type of information to which liberals and conservatives may respond differently is toxic content—defined as harmful, hostile, or aggressive language. It is assumed that conservatives have a relatively greater need for order and place greater value on tradition than do liberals (25). Toxicity violates social norms (26) and thus counters convention, order, and tradition. Along these lines, Mutz (27: 106) states, “Republicans and conservatives may be particularly sensitive to norm-violating threats to the social structure.” Mutz shows that negative emotional arousal is especially high among Republicans (and Independents) when exposed to toxicity (also see 28). This suggests they may be less inclined to share toxic communications given the negative emotions associated and the desire to not violate norms (and maintain order). This forms the second hypothesis, namely that, relative to liberals, conservatives will be less likely to retweet issues that contain toxicity (*Hypothesis 2*, also see 4, 29).^b^

In our definition toxicity is a type of offensive language that differs from hate speech that explicitly targets specific groups (30). And to be clear, toxicity differs from incivility that typically refers to more subtle (e.g. impolite) statements ([31]). In our analysis we focus on tone rather than statements that evoke discrimination and prejudice. Prior work makes clear that toxicity is invoked when discussing the other party (triggering virality) (13). We will test whether conservatives will be less likely to retweet Republican MCs’ tweets about Democrats than liberals to retweet Democratic MCs’ tweets about Republicans.^c,d^

Our paper proceeds as follows. First, we analyze the audience composition of Democrat and Republican MCs. Second, we consider policy issue engagement from both the supply (the perspectives of MCs) and demand (retweeting users) to assess H1. Third, we assess H2 by studying the sensitivity to toxicity from both supply and demand. Lastly, we build a gradient-boosting regression model to consider the overall effects for Democrat and Republican MCs.

## Data

We evaluate our hypotheses using data from Twitter (now X). While it is well understood that active users do not reflect the general population, behaviors on this or other social media websites provide insight into partisan decision-making in an important informational context (given its reach). Indeed, the incarnation of Twitter we use here (period between 2009 and 2019) no longer exists given changes to the platform (and its renaming). Yet, again, the data we employ provide a critical assessment of general partisan behaviors.

We use Frimer et al. (5) as the seed dataset (*N* = 1,293,753), which contains all tweets from members of Congress between 2009 and 2019, along with toxicity scores based on the *Google Perspective API*. Using this seed dataset, we used the official Twitter API to extract all retweets of MC tweets, and the timelines of all the retweeters. As explained, we focus on retweeting as indicating information diffusion phenomenon on the platform. As retweeting does not entail endorsement, we do not interpret retweeting solely as endorsement, though this may be the case for some tweets (32).

We label the members of Congress based on their party affiliation at the time, along with references to parties at the time. We implemented the same approach as Rathje et al. (1), using a list of keywords that refer to Democrats (such as left-wing, liberal, etc.), Republicans (right-wing, conservatives, etc.), and the 40 most popular politicians based on a list polled and maintained by YouGov (i.e. a reference to Obama). As examples of out-group toxicity, we show the top two tweets by toxicity from Democrats and Republicans:

- Maxine Waters (Democrat): “Calling Trump a moron was too complimentary. Let's ask Michael Che what he thinks!”
- Dana Rohrabacher (Republican): “@AOC my how intellectual of U. Guess U R so superior U can call those with whom U disagree assholes & still think highly of Urself.”

These are clear examples of out-group toxicity, where MCs of one party refer to the other party with high levels of toxicity. The full details can be found in the methods.

We additionally consider the following variables: policy topic (machine-learned labels with an accuracy of 88% based on the Comparative Agenda Project) and user ideology based on user timeline data (weighted score based on media domains tweeted). This measure of user ideology follows prior work (e.g. 33, 34) and, as we explain in the Methods section, avoids the circularity present in other strategies. The dataset contains a total of 10,451,080 retweet events, 1,435,132 unique tweeters, and 3,522,734,792 timeline tweets (used to estimate user ideology).^e^ We label audience members as liberals (Democrats) or conservatives (Republicans) based on their media diet over time. While we suggest this captures ideology (partisanship), an alternative (and perhaps less presumptive) interpretation is to think of it as a political diet.

We employ partisanship and ideology as proxies for one another, assuming that Democratic (Republican) MCs share an outlook and identity with liberal (conservative) users. We will use the terms interchangeably, noting the high degree of ideological sorting among partisans (e.g. 35).

## Results

**Fig. 1.**
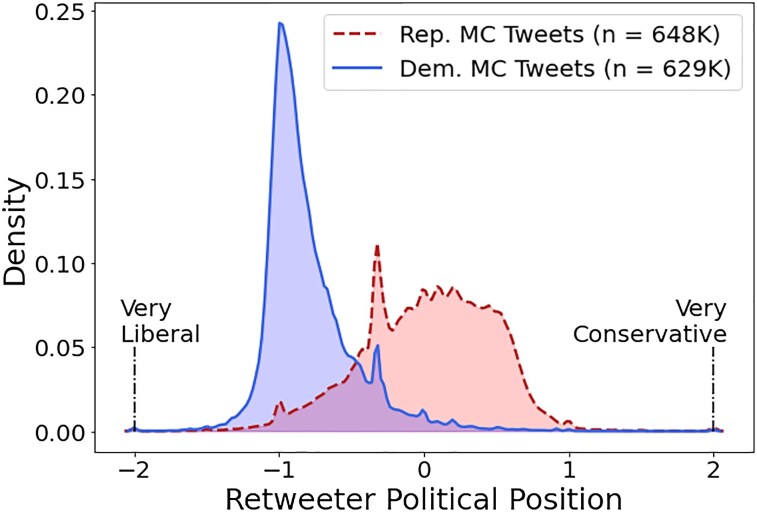
Density estimate of audience response by Republican MCs and Democratic MCs. Republican MCs garner a more diverse retweeting audience, which implies more liberal users tune in.

Figure 1 shows the ideology of those who retweet MCs’ tweets. The x-axis provides the political position of the retweeter with lower (higher) scores being more liberal (conservative). That is, negative values (between −2 and 0) indicate a liberal/Democrat-leaning user, while positive values (between 0 and 2) indicate a conservative/Republican-leaning user. The y-axis provides the probability density estimate, with the solid blue line indicating Democratic MCs and the dashed red line Republican MCs. The figure reveals that for Democratic MCs, the retweeters are much more likely to be homogeneously liberal; in contrast, for Republican MCs, the retweeters are much more likely to be widely distributed, including a nontrivial proportion of liberals (i.e. only a slight skew to the right). This implies liberals are more likely to tune in to Republican MCs than conservatives to Democratic MCs (also see 2, 36). This coheres with the idea that liberal retweeters share from across both sides of the aisle while conservative retweeters are, relatively, more restricted to Republican MCs. When we more explicitly test our predictions, we find a Mann–Whitney *U*-statistic of 204,527,680,094 and *P*-value of <0.01. We also check this distribution and subsequent results using gold-standard DW-Nominate scores as Fig. S6 in the Supplementary Information (3, 37).

### Policy engagement

Our first hypothesis states that conservatives will be less likely to retweet Democratic MCs than liberals to retweet Republican MCs. The hypothesis rests on the assumption that conservatives avoid engaging with the liberal policies (that Democratic MCs likely discuss) more so than liberals avoid conservative topics (that Republican MCs likely discuss). To identify issues, we trained a supervised machine learning classifier using labeled tweets from Russell (38), with the assumption that the linguistic characteristics of tweets between Representatives and Senators are similar. The dataset includes a total of 68,398 tweets, 45,402 of which are labeled with codes from the Comparative Agenda Project's (CAP's) codebook (39) and 22,996 labeled as nonissue related tweets. We implemented a variant of the large BERTweet architecture, a pretrained model from Nguyen et al. (40), and trained two classifiers—one for multiclass classification of 20 topics and one for binary classification to see if a tweet relates to policy or not, distinguishing if policies are mentioned. Our first (multiclass) model produces an accuracy of 86.4% for classification across all 20 policy topics (from CAP). Our second (binary) model produces a score of 88.5%. This outperforms the current best model at 79% (41). Methodological details are further discussed in the Methods section.

**Fig. 2.**
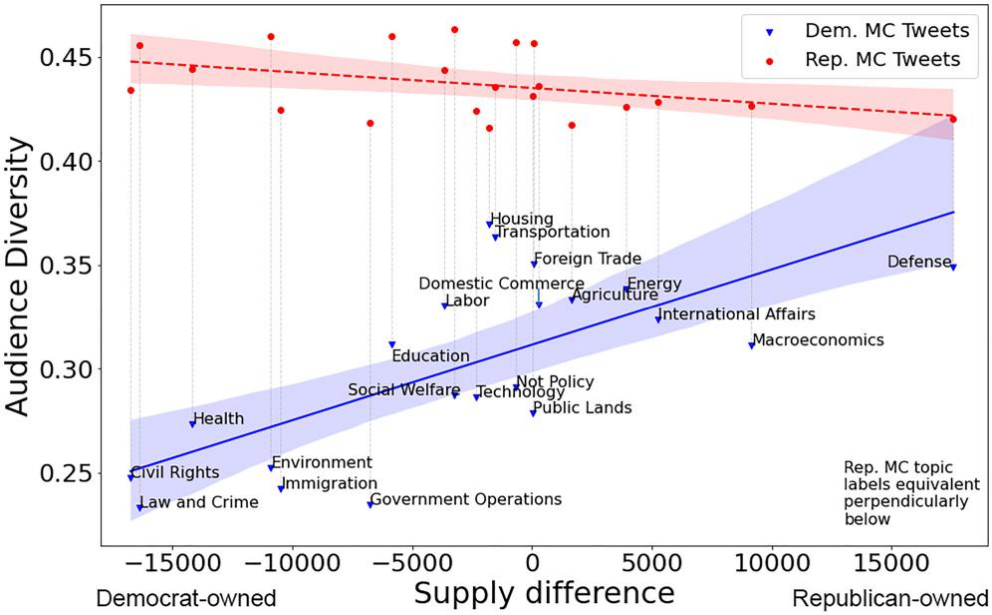
Policy issue items versus audience diversity. Blue points indicate tweets by Democrat MCs; red points indicate tweets from Republican MCs. The X-axis denotes the supply difference of Democrat and Republican tweets about a certain policy issue—negative values indicate Democrat MCs tweet more and positive values indicate Republicans tweet more. As shown by the greater diversity of the red line, Republican MCs elicit greater response from liberal users than do Democratic MCs from conservative users. The positive slope of the blue line indicates, when Democratic MCs tweet about more “conservative” issues (e.g. defense, macroeconomics), diversity increases. When Democratic MCs tweet about more “liberal” issues (e.g. civil rights, the environment), diversity is low. Republican MCs have high audience diversity across all topics, but a slight negative slope indicates increased engagement of liberals when Republicans tweet about Democrat-owned issues. Democrat cross-cutting sharing is overall higher. Republican topic labels are equivalent to Democrat labels vertically below.

We then classified issues as liberal/Democratic or conservative/Republican based on their relative supply. In Fig. 2, the x-axis shows the difference in tweet supply for each policy (“Republicans minus Democrats”). This operationalizes right and left agenda items. Positive values indicate Republican MCs tweet more; negative values indicate Democratic MCs tweet more. The y-axis measures political diversity via the standard deviation of political position of the audience (i.e. retweeters). This reflects political diversity for the given topic (the results are *not* affected by the frequency with which a given topic is tweeted by MCs). Blue dots are aggregate tweets from Democratic MCs and red dots from Republican MCs. The figure shows that Democratic MCs tweet Democratic topics, including civil rights, health care, environment, immigration, and education, while Republican MCs tweet Republican topics such as defense, macroeconomics, and international affairs. For the most part, these results align with prior work on issue ownership that posits that partisans emphasize issues on which voters seem to think they perform well (e.g. 17; [42–44]). (The main exception issue is Democratic MCs’ relative emphasis on law and crime despite it being a traditional Republican issue. This likely reflects Democrats using the terms “criminal” or “crime” to discuss Trump during the Russia probe, which would inflate the supply of this topic for Democrats.)

The figure provides strong support for Hypothesis 1; when Republican MCs tweet, the audience diversity is always very high (consistently around 45%, with a slight downslope). In contrast, when Democratic MCs tweet on issues usually owned by liberals (i.e. civil rights), the diversity, meaning cross-cutting diffusion, is low. Moreover, when Democratic MCs tweet about issues usually owned by conservatives (i.e. defense), diversity is high, which suggests that cross-cutting diffusion is high. Liberals consistently share Republican MCs communications across policy topics, whereas conservatives are more selective and only share Democratic MCs’ messages on conservative issues. To further ensure the validity of the audience variance, we also plot the average political position (Fig. S1 in the Supplementary Information), which shows that the direction of diversity does indeed come from the out-group. This provides a useful confirmation, since our definition of liberal and conservative issues is based on the supply difference (see Table S2 in the Supplementary Information); it also shows that the MCs of a given party pursue the strategy of emphasizing their own issues. Replication of results using Nominate scores are found in Fig. S7 in the Supplementary Information.

**Fig. 3.**
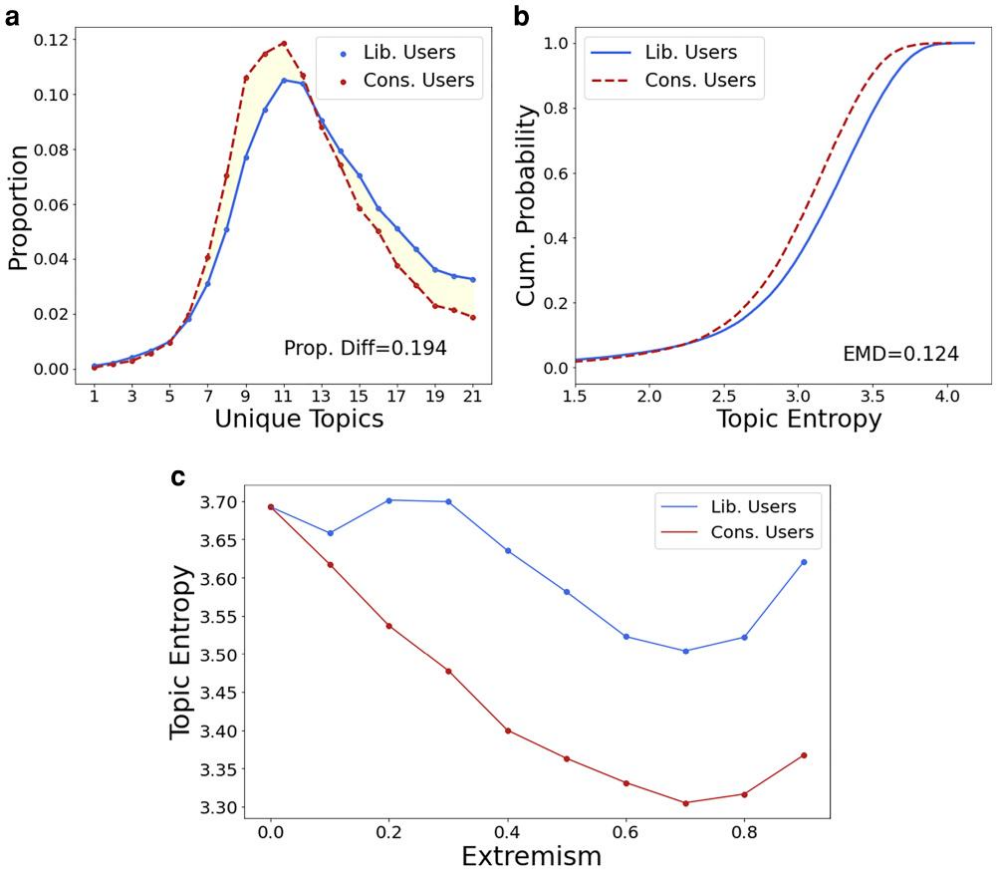
a) Unique policy topics retweeted, b) aggregate measures of entropy (the more left the sigmoid curve, the more diversity), and c) topic entropy against ideological extremism with LOESS. It appears that liberals are exposed to and share with more policy topics than do conservatives. Also, topic entropy decreases with extremism until a pullback at higher levels of extremism.

While Fig. 2 shows results from the MC's perspective (demand side), Fig. 3 shows the greater diversity of liberal users directly. Figure 3a shows the user distribution of unique topics, filtered on users who retweet at least 21 times (users who can possibly engage with all topics). The x-axis represents the number of unique topics which a user has shared, and the y-axis shows the proportion of users who engage with that specific number of unique topics. The heavier right tail of liberals shows that there is a greater proportion of liberals who share more diversely. This yields an absolute proportional difference of 0.194, which is the signed difference between the two curves. Additionally, any liberal who retweets more than 10 unique topics will be guaranteed to tweet a Republican-owned topic by the pigeonhole principle, and according to Fig. 2. Therefore, liberals share more diversely.

This, however, does not account for distributional skew within the topic categories. For instance, a liberal user can share all twenty topics, but Democrat-owned topics multiple times and Republican-owned topics just once, each, which is heavily concentrated on one end. For every user, we thus attach a score for topic diversity based on defining a Shannon entropy:


H(x)=−∑p(x)logp(x).

Here, *p*(*x*) denotes the probability of an event (in our case, the frequency), while log *p*(*x*), denoted the information content. Thus, a higher Shannon entropy in this context means greater diversity. Figure 3b shows the cumulative distribution, where the x-axis denotes diversity and y-axis denoted the cumulative frequency. The more rightward the S-shaped curve is, the more diverse that group is. This demonstrates, once again, that liberals share more diversely. This divergence can be quantified in three ways. First, the Earth Mover's Distance quantifies the distance between two CDF comes out to be 0.124, which would correspond to a maximum shift of 0.52 entropy. Second, the Kolmogorov–Smirnov (KS) Divergence test yields a KS-statistic of 0.174 and *P*-value of <0.01, demonstrating statistical significance. Third, the extent of the partisan difference is sizable, captured by the yellow shading in Fig. 3a. When aggregated, it is equivalent to a 19.4% divergence, namely where liberals share more diversely than conservatives. Put another way, liberals retweeted 19.4% more diverse topics than did conservatives (a ratio of 1.19). We validate these findings using a more extreme cutoff, namely the median (−0.856) to define liberals and conservatives, which increases the definition of conservatives to include more moderate users around 0. This actually increases the divergence, which serves as a robustness test and that moderate users are less diverse in issue sharing (Fig. S2 in the Supplementary Information; replication of results using Nominate scores are found in Fig. S8).

We can further show that this scales with ideological extremism. Figure 3c shows that as we scale the absolute value of ideology, topic entropy decreases using LOESS smoothing (Locally Estimated Scatterplot Smoothing, which is a non-parametric regression method that fits weighted least squares regressions to localized subsets of data to create a smooth curve), and that this happens faster for conservatives. This coheres with a distinct, albeit not exclusive, perspective, namely those with more extreme ideologies on either side will behave differently than their less extreme counterparts. Here, it could be that more extreme ideologues tend to rationalize their standing perspectives to a greater extent (45), and as such, may be less willing to pass along content on a wide array of issues. In this sense, homogeneity increases with extremism, and conservatives exhibit this to a greater extent, shown by the lower average. The compounding of homophilous ties and lower topic diversity for these intermediaries could have downstream effects for who generates virality.^f^

### Toxicity

**Fig. 4.**
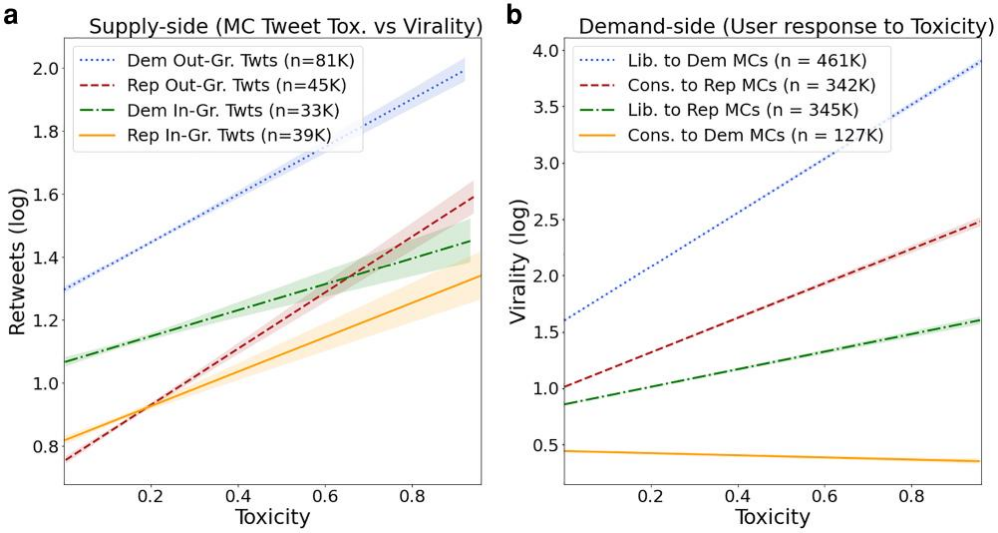
Virality versus toxicity, split by a) partisan in-/out-group mentions and b) user-level response. Out-group tweets are generally more viral and toxic (a). Republican out-group tweets can be viral (red)—due to response from the left (a).

We next turn to group status that is invoked within the tweets, regardless of issues. As mentioned, an out-group tweet occurs when an MC refers to the other party; and in-group tweet occurs when an MC refers to their own party. Figure 4a displays the relationship between the toxicity of an MC's tweet about their or the other party and the extent to which the tweet is retweeted. Each observation is a tweet. The figure shows, consistent with prior work, that in every case, as toxicity increases, so does retweeting (e.g. 4). It also shows that Democratic MCs’ out-group tweets that are particularly toxic are most likely to be retweeted. In contrast, Republican out-group MCs’ tweets have a much lower level of retweeting. Figure 4b plots different audience responses to distinct tweets by MCs. It shows that liberals generally retweet much more toxic tweets than do conservatives. Indeed, conditioning upon the partisan source, the graph shows that the gap between users’ reactions grows as toxicity increases (the slopes on the liberal user lines are much steeper than those on the conservative user lines). Substantively, based on the ratio of slopes, liberals are 1.56 times more sensitive to toxicity from their own party than did conservatives. Moreover, liberals retweeted toxicity from the other party 8.30 times more, where crucially, a downward slope for conservatives (yellow) indicates as Democrats increase the toxicity in tweets, conservatives engage less. We further classify a tweet as toxic if its toxicity score is greater than the mean plus one standard deviation (0.237), then run a test of proportions on both the supply and demand (raw counts are shown in Table S5). Our hypothesis 2 is that Democrats tweet and liberals retweet more toxicity. Both yield *P*-values less than 0.001, with the supply Z-statistic of 90.9 and demand Z-statistic of 213.2. The full coefficients and residual tests are included in Table S6 of the Supplementary Information.

In summary, retweeting occurs much more when Democratic MCs create toxic content about Republicans than vice versa, and this reflects that liberals are more likely to retweet toxic tweets. This is consistent with Hypothesis 2. Liberals will retweet toxic critiques of Republicans, but conservatives are less likely to retweet toxic tweets of Democrats, from out-group elites. This aligns with each MC's incentives for maximizing virality. Therefore, liberals share (i.e. retweet) more diverse issue agendas, more discussion of the other side, and more toxicity. In short, the diversity displayed in the audiences for MCs from different parties (Fig. 1) arises from the divergent ideological behavior based on policy issues and toxicity.

### Supply side

It is useful to explore whether supply-side effects drive any of the variations in liberal and conservative sharing (e.g. 5). In Table 1, we present confusion matrices of MC tweets by nontoxic/toxic and not policy/policy content, split by party. The bottom row shows the ratio of toxic to nontoxic tweets per that policy category. As above, we classify a tweet as toxic if it is greater than the mean plus one standard deviation (0.237). These results remain the same when using the mean (see Table S4, the additional standard deviation is more conservative). The main finding can be captured by comparing the nontoxic/not policy category versus the toxic/policy category. For Democratic MCs, the respective percentages are 32 and 12%, whereas for Republicans they are 41 and 7%. In short, Democrat MCs tweet much more about policy, and they do so with much more toxicity than do Republicans. The overall toxicity is 15% for Democratic MCs and 10% for Republican MCs. This suggests that the supply of content differs between partisan MCs.

**Table 1.** Policy versus nontoxic/toxic content, across Democratic and Republican MCs.

	Not policy	Policy	Not policy + Policy
(a) Democratic MCs			
Nontoxic (%)	32.3	52.6	84.9
Toxic (%)	3.3	11.8	15.1
Nontoxic/Toxic ratio	9.739	4.447	—
(b) Republican MCs			
Nontoxic (%)	41.4	48.7	90.2
Toxic (%)	3.1	6.7	9.8
Nontoxic/Toxic ratio	13.25	7.27	—

Democrats tweet about policy with more toxicity than Republicans (4.4 versus 7.3 civility ratio).

We next look at the relationship of the number of shares with MC choices. To do so, we train a gradient-boosting regression model, which can capture nonlinearities in variable interaction. We regress the number of retweets (i.e. virality) per MC party, using the following variables:

- Policy difference: The supply difference of the 20 Comparative Agenda Project's Policy Topics (see footnote a).
- Policy: Whether it is a policy issue or not.
- Toxicity: Toxicity level of tweet.
- Group status: Whether a tweet is in-group, out-group, or not related.

We then employ Shapley-explainers to measure the impact each variable has on the overall virality (see Methods). This allows us to identify the extent issue ownership, toxic language, and group status have on the virality of MCs, and assess divergences in systems-level diffusion of their content. As we do not know when a user sees a tweet (exposure), tweets themselves have to be the unit of analysis.

**Fig. 5.**
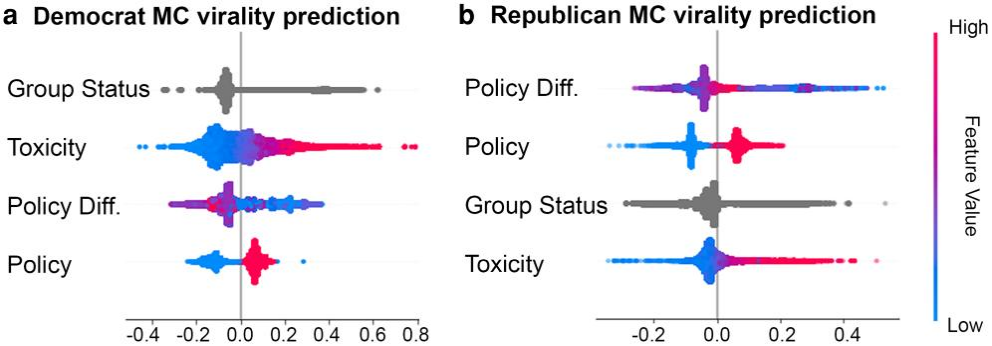
CatBoost regression on the four variables, then ranked top to bottom in terms of feature importance, separated by MC tweets (a) Democrats and b) Republicans). We find toxicity and group status more predictive for Democratic MC tweets and policy topic more for Republican MC tweets.

The results are shown in Fig. 5. We find that Democrat MC tweets become much more pervasive (viral) when they invoke the other group and when they are toxic. In contrast, Republican MC tweets are shared more when the policies are “their” policies (hence less overall diversity). More generally, since we find Democrats MCs tweet with more toxicity that draws them more of an audience. This makes sense given liberal users are found to be relatively drawn to toxicity. Details on training and error are reported in Table S7 in the Supplementary Information.

**Fig. 6.**
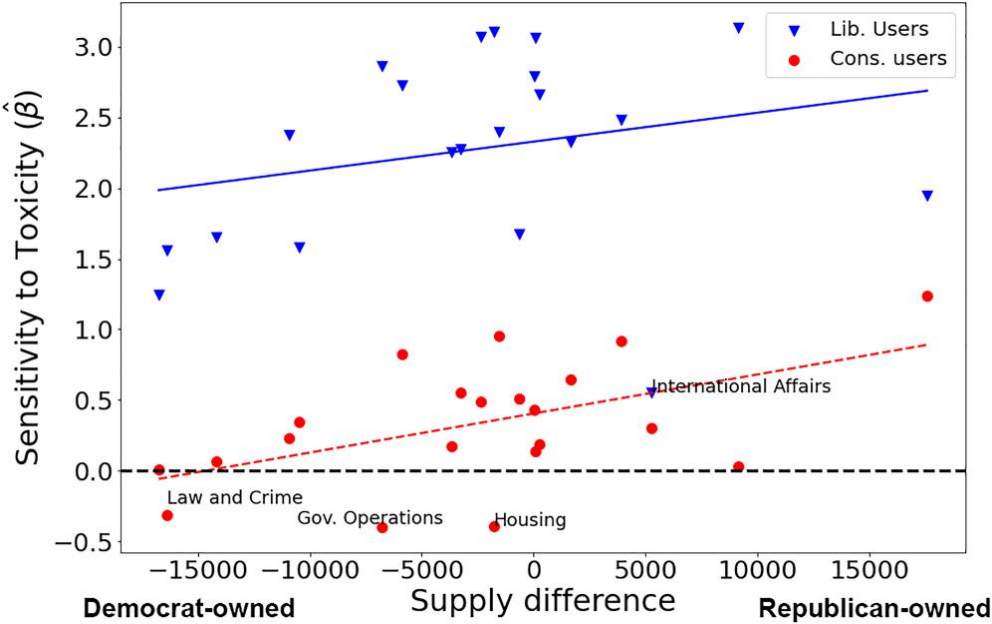
Partisan agenda items versus sensitivity to toxicity (slope of toxicity to virality), broken down by liberal and conservative users. Democrats seem to discuss international affairs with less toxicity. Labeled dots are explicitly discussed.

In Fig. 6, we further look at the demand for toxicity. Here, we plot the effect of toxicity on retweeting on the y-axis (i.e. the regressed slope from virality on toxicity; tabular results shown in Table S3). This is a proxy for sensitivity (i.e. y = 0 indicates no change to virality based on toxicity). The x-axis shows the ideological dispersion on the given issue with each dot indicating an issue tweeted by a Republican MC (red) or a Democratic MC (blue). In line with what we have already shown, when toxicity is used in a tweet, liberals are substantially more likely to retweet it than are conservatives. The figure reveals a large effect. On Democratic issues, when there is toxicity, conservatives in fact are less likely to retweet it (lower beta/sensitivity). Even on Republican issues, they retweet toxic tweets at much lower rates than liberals do. There are no issues that conservatives are more likely to retweet when they are toxic, though this reflects somewhat that conservatives retweet less and also spend less time on Twitter. Republican MCs gain the attention of liberals for cross-cutting issues through toxicity (without compromising their in-group followers), but not vice versa.

## Discussion

We investigated whether there are aspects of social media where liberals and conservatives spread information differently. We tested for variation on Twitter, the only available social media data that provide the demand-side measures. Our results provide clear evidence that, at least in this general type of interactive social media domain, liberals and conservatives choose to pass along different types of information from political representatives to other users. Specifically, we show how information environments result from both supply and demand preferences.

We have three main findings. First, Republican members of Congress have a more diverse audience than Democrat MCs, implying that liberal users share issues emphasized by Democrats and Republicans, while conservative users pass along information mostly from Republicans. Second, conservative users are more selective about which policies they retweet, avoiding topics that are not on the conservative agenda. This is consistent with work showing socially driven (but not necessarily news-driven) policy bubbles (e.g. 3; 46, 47). Ours is one of the few analyses, however, to document the liberal and conservative differences in the nature of those networks (although, see Ref. (2 for tie-level homophily).^g^ Third, toxicity creates different patterns of diffusion: liberals retweet toxicity more than conservatives do.

Put together, our results show that if the forces that generate diffusion for one party diminish sharing for the other, any form of algorithmic intervention such as toxicity filters may results in one group sharing much less information. Specifically, liberals and conservatives react differently to information and, consequently, rebroadcast distinct types of information. Given at least some tendencies toward homophily in exposure (11), an implication is that liberals will view a host of policies and toxicity passed along from other liberals, while conservatives will mostly see issues on the conservative agenda and relatively less toxicity passed along from other conservatives. Wojcieszak et al. (36) find that most Twitter users do not follow partisan elites, which indicates a diffusion gap between partisan elites and the general public. It is plausible, if not probable, that most users receive more political information from other users via retweets. In that sense, the individuals we studied here serve as intermediaries as per the canonical theory of two-step information flow, where the general public receives political information not directly from elites but secondhand, from other citizens (e.g. 49–54).

While the composition of the intermediaries’ audiences is not something we measured, there has been extensive work detailing the extent of homophily across liberal and conservative lines on Twitter. Boutyline and Willer (2) find that conservative and ideologically extreme individuals exhibit greater levels of homophily and posit that this is caused by a stronger “preference for certainty.” Putting this together with our finding that liberals retweet a more diverse array of topics suggests that this diversity is compounded with liberals’ more heterophilous social ties. Conversely, conservatives sharing less diverse and homophilic ties likely creates an “information bubble.” Liberals thus are more likely to engage with heterogeneous content and with heterogeneous sources, relative to conservatives. Moreover, the compounding effects of toxicity, tie homophily, and topic diversity mean the demand for out-group toxicity may not necessarily arise from their expected constituents.

The conclusion is that there are very distinct information environments for liberals and conservatives, with liberals being exposed to a wider array of policy topics but also more toxicity relative to conservatives. These are akin to polarized information bubbles since the information ecologies in which liberals and conservatives end up are very different from one another. This type of polarization (i.e. where there is a large gap between groups; (55) is not the same as ideological polarization or affective polarization; here polarization refers to information content. Nonetheless, that content could contribute to other forms of polarization. For instance, since ideologues tend to have more extreme positions on their own policies (i.e. conservative positions on conservative policies) (56, 57), a homogenous policy bubble could increase ideological polarization. Alternatively, an information bubble that envelopes toxicity from the other party (e.g. liberals engaging with conservative-generated toxicity) could make them more affectively polarized (28). More generally, these bubbles likely make cross-ideological or cross-partisan interactions and perspective-taking more difficult since those with distinct outlooks engage from very different places.^h^ Finally, our findings clarify downstream implications for how social media should be designed, if for the purpose of increasing exposure and engagement with outgroups. Symmetric approaches, such as blanket thresholds for toxicity or out-group topics, would have heterogeneous success based on the ideology of the user.

One possible limitation of this study is the possible effects of algorithmic amplification. As Huszár et al. (58) showed, Republican MCs were amplified more than Democratic MCs, which may cause downstream effects in what users see, and therefore subsequent engagement. However, in the absence of the number of views—which was only made available after data collection was made—and also by whom, it is not possible to observationally disentangle the engagement conditioned on exposure. Moreover, as Munger (59) states, the logic of algorithms changes day-by-day. The algorithm that drove the 2016 elections could be substantially different than what was measured in 2020. In a similar vein, the “while you’re away” feature, introduced on Twitter in 2015, showed posts that a user might have missed (60); only in 2016 did algorithmic curation enable posts in reverse chronological order (61). Since our dataset covers 2007 to 2019, more than 70% of the data captures organic engagement (i.e. prior to algorithmic curation), and covers five full Congresses. Regardless, as with all social media studies, further exploration over-time is important. Additionally, as with many country-specific studies, additional research is required to ascertain the level of generalizability. The phenomenon we observe here may potentially be caused (or exacerbated) by the notably high overlap of ideology and partisanship in the United States’ two-party system (35), thereby driving the ideological asymmetric information bubbles.

Other limitations may provide the blueprint for future work. First, we did not consider factors such as whether tweets contain URLs, media, or moral language–variables known to increase virality. This is because our principal interest is in system-level divergences in conservative and liberal information environments, arising from group and policy differences, rather than message-level effects. Even so, explaining the impact of these content related variables is an important topic for future work. Second, we did not consider control for audience size, which would be necessary for investigating message-level effects. This is a general limitation of longitudinal analyses, as these data are unavailable: we cannot account for the audience *at the time* of tweeting. This makes models such as logistic mixed models not possible, as we do not know when a user sees a tweet but does not retweet it. As such, tweets themselves have to be the unit of analysis. Future research that includes exposure data may alleviate this weakness. Lastly, the longitudinal nature of the analyses makes clear that future work would benefit from more explicitly exploring temporal dynamics. Our hope is that our work provides a foundation for these and other investigations.

## Methods

We extend Frimer et al. (5)'s data source to include the following covariates (at the MC tweet level, i.e. the supply level): CAP topic, in-/out-group labeling, MC source party labeling, and, upon scraping retweeter timelines, user ideology labeling. We use the toxicity scores provided by Frimer et al. (5) (which are based on an independently validated operationalization of PerspectiveAPI's “toxicity”). The full data descriptions can be found in Table S5 in the Supplementary Information.

**Topic (Supervised):** We trained a supervised machine learning classifier using labeled tweets from Russell (38), with the assumption that the linguistic characteristics of tweets between the House and the Senate are similar. The dataset includes a total of 68,398 tweets, 45,402 tweets labeled with codes from the Comparative Agenda Project's (CAP's) codebook (39) and 22,996 labeled as nonrelated tweets. The dataset also overlaps with our dataset.

The best models from Russell (38) feature F1-scores of 79% and include augmentation via the Linguistic Inquiry and Word Count (LIWC) scores. LIWC is a gold-standard package for computerized text analysis covering a range of psychological and topical categories and social, cognitive, and affective processes (62). Using these features, we implement deep learning, specifically a variant of BERT (Bidirectional Encoder Representations from Transformers), which is a variant of the transformer architecture fine-tuned for the English language.

We implement the large BERTweet architecture (a specialized BERT model for Twitter and tweets), using a pretrained model from Nguyen et al. (40). We train two classifiers—one for classifying the 21 topics (20 CAP topics and nonpolicy tag) and another for binary classification of if policies are mentioned. Our first (multiclass) model produces 86.4% accuracy for classification across all 20 policy topics. Our second (binary) model yields 88.5% accuracy, both of which outperform those by Russell. Full classification results are given in Tables S1 and S2 in the Supplementary Information.^i^

**Group labeling:** We implemented the same approach as Rathje et al. (1), using a list of keywords that refer to Democrats (such as left-wing, liberal, etc.), Republicans (right-wing, conservatives, etc.), and the most popular politicians based on polling from YouGov.

**Source party labeling:** A total of 831 members of Congress had Twitter labels and were labeled. Several have changed accounts, and these were manually merged. For those who have switched parties, their party affiliation at the time of the tweet was used.

**User ideology:** Ideology scores were labeled based on a user's timeline history (up to 3,200 tweets per the limit of the API). This yielded a total of 3,522,734,792 tweets. URL domains were extracted from the timeline. Associated with each URL is a political score of [−2, −1, 0, 1, 2], corresponding to “left,” “left-center,” “center,” “right-center, and “right,” based on Media-bias/fact-check. A weighted political score is then calculated based on the proportion of tweets from each category. Only tweets prior to a retweet are used to measure ideology to allow for the possibility that there is a retweeter switch—where a user retweeted more left-leaning content, but later switched to more right-leaning content.

There are a few established methods for computing user ideology. In particular, Barberá's (63) Bayesian point estimation is particularly popular in political science research, along with roll-call votes-based estimates like DW-Nominate scores (64, 65). It relies on the network of following users and public political figures on Twitter, and their affiliations, to generate an ideological score. However, as the principal direction of our analysis is the dyadic ties between retweeters and MCs, this creates significant circularity as who people follow will dictate their information environments. In other words, the ideology score would contain the outcome. In contrast, the media diet approach has been adopted in many contexts related to Twitter ideological modeling (5, 7, 33, 58, 66). There are two interpretations. First, it is an absolute measure based on the bias of media outlets. Second, it is a relative measure based on everyone else's diet. We show our results are robust across both the absolute threshold of 0 and the median. For topic modeling, we also considered topic modeling approaches such as BERTopic and Latent Dirichlet Allocation (LDA); however, relying on the CAP classification allowed us to directly answer our theoretically driven questions.

## Supplementary Material

pgaf206_Supplementary_Data

## Data Availability

The seed data in this article can be found in Frimer et al. (18). The code and processed data can be accessed at this GitHub repository: https://github.com/herbertfreeze/Ideological_asymmetries.

## Notes

a This is consistent with Barberá et al.’s (3: 1537) study of behavior on Twitter. Barberá et al. (3) rely on a grounded approach arising from temporal events (e.g. the Boston Marathon bombing). In contrast, as we will explain, we use established criteria for topic ideology based on the Comparative Agenda Project. The Comparative Agendas Project (CAP) is a global research initiative that systematically tracks and analyzes policy activity across different countries and political systems. CAP originated from the US Policy Agendas Project, developed by Frank Baumgartner and Bryan Jones. We also note that there is contrary evidence that conservatives engage more in cross-cutting conversation on YouTube (24). The difference between Twitter and YouTube may reflect that the former involves passing along information that could generate a response whereas the latter is a unilateral experience.

b Theocharis et al. (29) and Frimer et al. (5) focus on toxicity from a supply perspective whereas we focus on a demand perspective.

c Rathje et al. (1) report no difference by the ideology of members of Congress or media outlets; however, they focus on differences in sharing out-group messages (or in-group messages) against messages that do not invoke either group at the supply-level. Our focus is on audience response (demand-level) analysis for understanding engagement with the other side. Distinctly, we have avoided referring to our expectations in terms of ideological symmetry or asymmetry given we do not offer a direct psychological test of underlying belief systems.

d Recent Pew Research shows that more Conservatives (compared to Liberals) believe that many people take hateful content too seriously. This would suggest, if our hypothesis is accurate, that conservatives may be more sensitive to toxicity but at the same time view it as a less serious problem.

e Our dataset differs from Rathje et al. (1) primarily in timespan, including MC tweets from between 2009 and 2019. Notably, this then includes early online discussion of the 2016 primaries (which does not seem to be as present in Rathje et al. (1), whose data are from 2016 to 2020).

f An extension is to explore the relationship between bots and media diversity, as bots may share a select few media sources by design. As they share in higher volumes, they may be over-represented at certain extremity bins, and due to their biases toward certain topics, skew topic diversity scores (32).

g Relatedly, Eady et al. (48) find that conservatives follow more politicians across the aisle. A key difference is they focus on a user subset based on follows, which is not required for retweeting. Our dataset also begins from the supply level, whereas they start from the demand level.

h Further, what appears to be a symmetric increase in virality to toxicity (1) is actually driven by asymmetric reactions in the demand, where liberals react to Republican toxicity but not the other way around.

i We considered topic modeling using BERTopic and LDA; however, given the high scores from BERT, it allowed us to most directly answer our theoretically driven questions.

## Preprints

This manuscript was posted on a preprint: https://osf.io/preprints/osf/x59qt.
