# Supplementary material for: Liberals and conservatives share information differently on social media
Source: PNAS Nexus. 2025 Jun 27;4(7):pgaf206. doi: 10.1093/pnasnexus/pgaf206 (PMC12280873; doi:10.1093/pnasnexus/pgaf206)
Supplement: pgaf206_Supplementary_Data [file pgaf206_supplementary_data.docx]

**Supplementary Information for**

**Liberals and Conservatives Share Information Differently on Social Media**

Figures. S1-8

Tables. S1-7

*
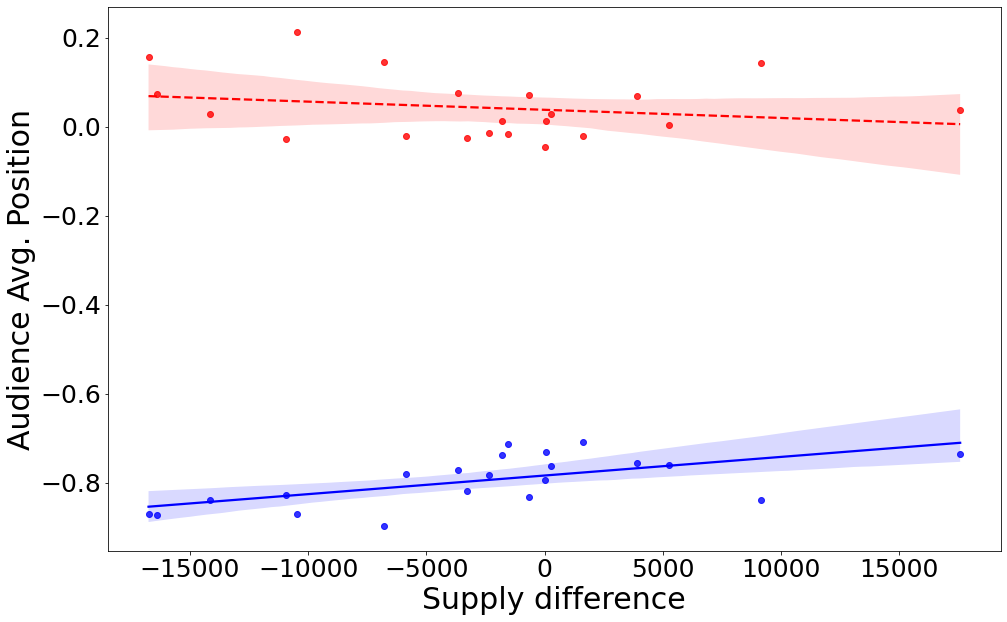
*

**Fig S1:** Average political position of audience, by topic and source partisanship (Party of the MC). Validates “starting points” for diversity values in Figure 2.


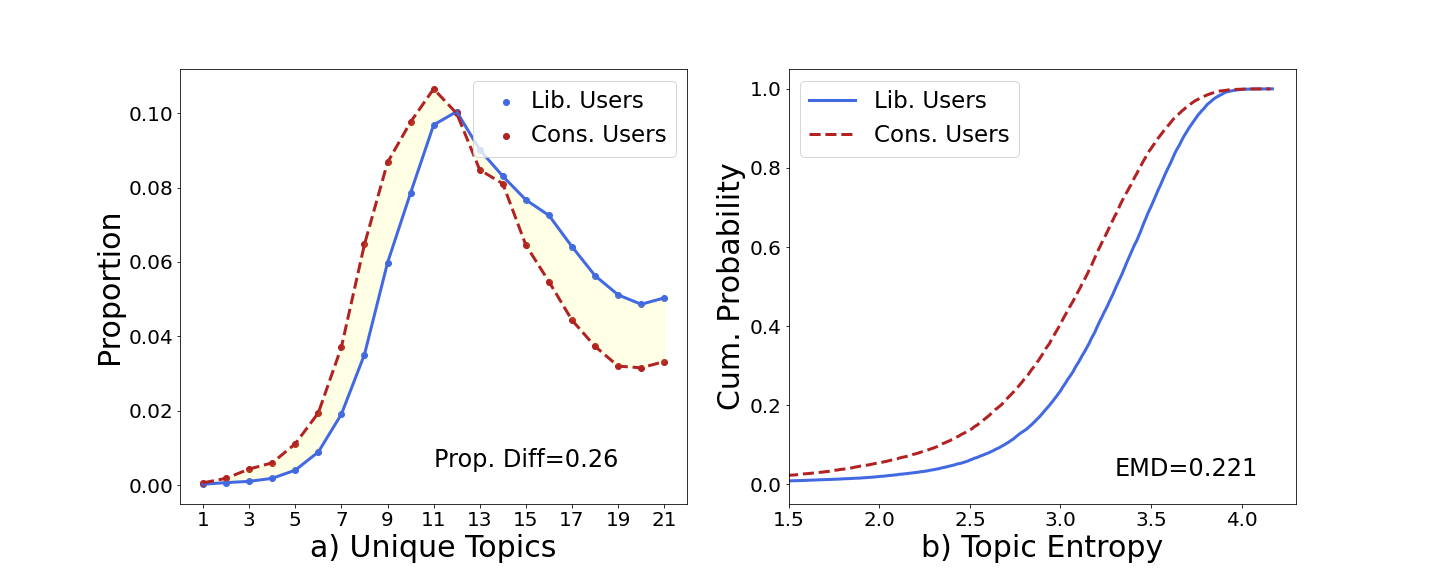


**Fig S2**: Equivalent to Figure 2 but with a median (-0.856) cut-off for liberal and conservatives. Liberals are exposed to and share with more policy topics than do conservatives, shown through (a) unique policy topics retweeted and (b) aggregate measures of entropy (the more left the sigmoid curve, the more diversity)


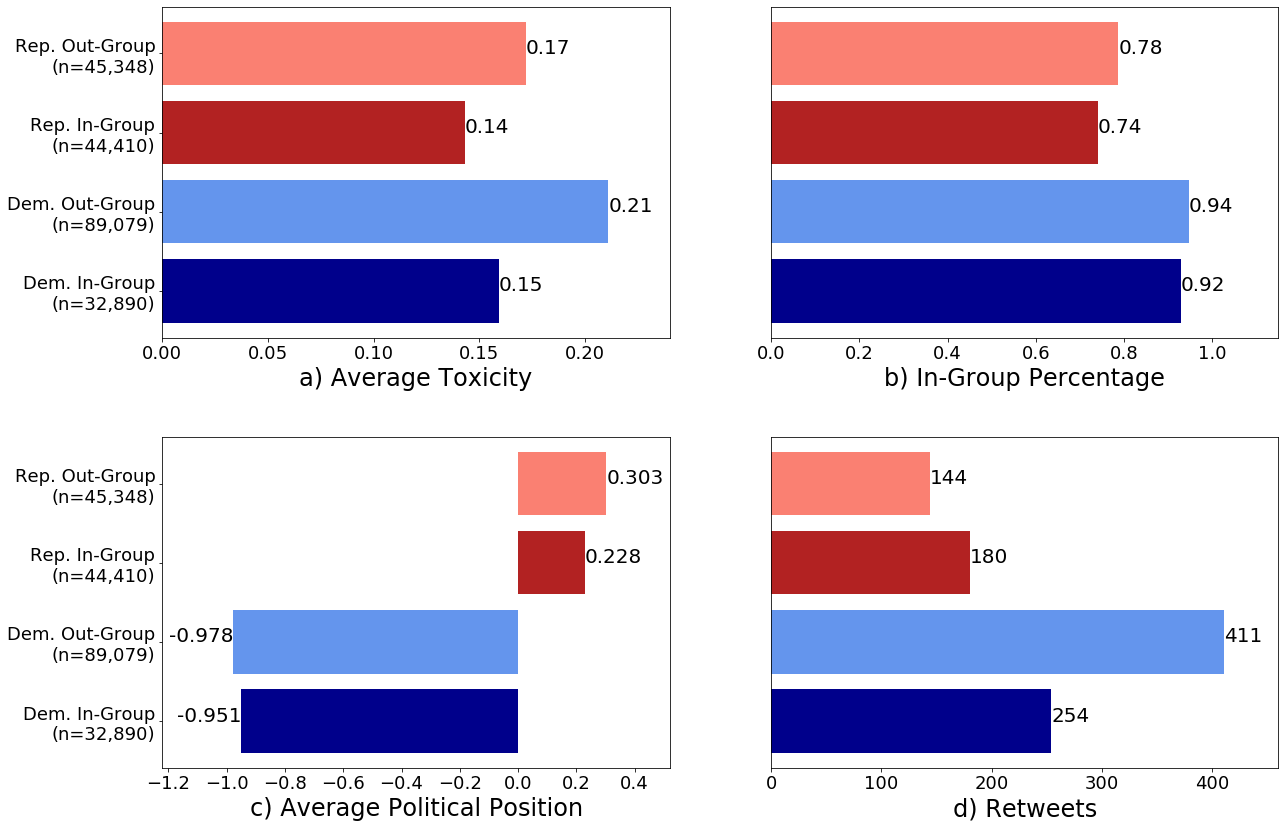


**Fig S3:** In-/out-group effects on (a) toxicity, (b) in-group composition, (c) political position, and (d) virality. Figure S3a reports the average amount of toxicity in distinct types of tweets, showing that tweets about the out-group from either party contain more toxicity. Figure S3b and c describe the profile of retweeters in terms of ideology, whereas Figure S3d displays the aggregate amount of retweeting. Figure S3b shows in-group audience composition of retweets, revealing—consistent with the diversity dynamic—that Democratic MCs are overwhelmingly retweeted by liberals, while Republican MCs have a smaller in-group retweet percentage. Figure S3c, which shows Democratic MCs get retweeted by an extreme set of liberal users, while Republican MCs draw only a moderately conservative set of users. Looking specifically at out-group tweets, we see that the ideology of the audience is more extreme for out-group tweets from both parties. Out-group tweets are more toxic, homogenous, and extreme. Figure S3d reveals that Democratic MC outgroup tweets get the most retweets, reflecting that liberals are much more likely to retweet such communications (which tend to be toxic). Republican MC in-group tweets are more viral than their out-group tweets. This reflects conservatives’ relative toxicity aversion (where toxicity is more present in out-party tweets) and the presence of Trump in the 2015 primaries and 2016 election driving more in-party retweeting.


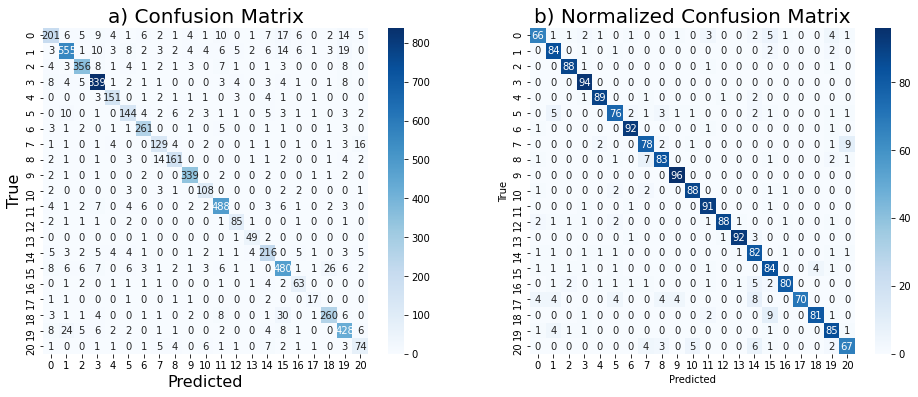


**Fig S4:** Confusion matrix of the classifier with a) raw counts and b) percentages.


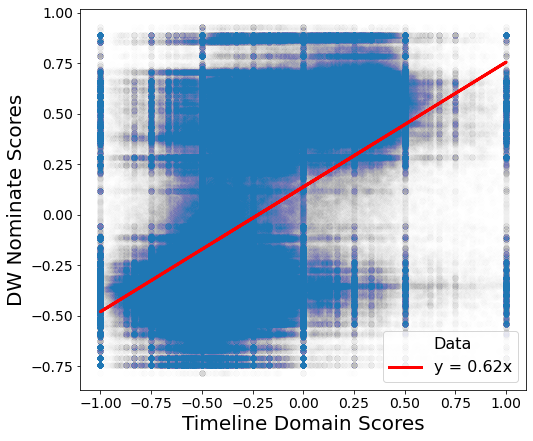


**Fig S5: Correlation between media diet scores and DW nominate scores.** We find a correlation between our estimates is 0.62, with categorization agreement of 75.2% Some users who are labeled as liberals may be moderate conservatives based on nominate scores, though this is mitigated by our other robustness check by using the median rather than mean (i.e. Fig. S2).


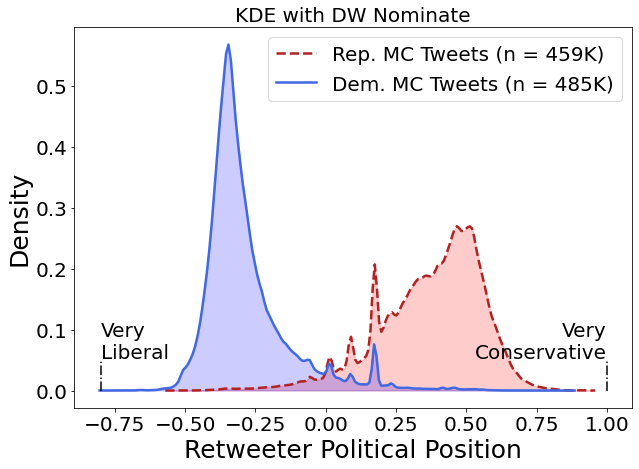


**Figure S6:** The distribution of ideological scores based on DW Nominate. The shape corresponds closely with Fig. 1, with conservatives more conservatives (right-wise translation). This is consistent with our hypothesis that the most popular media (i.e. NYT and Washington Post) lean left-center.


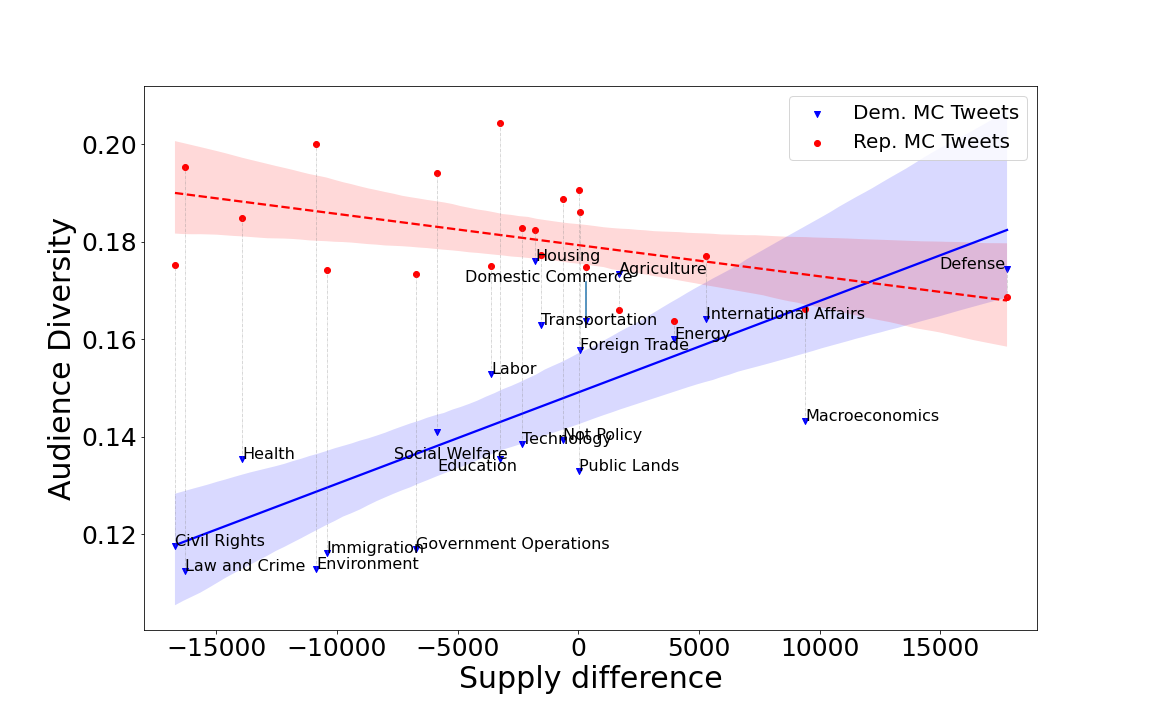


**Figure S7: Replication of Fig. 2 using DW Nominate scores.** DW nominate results strengthen our findings. We find a similar dynamic in audience diversity across Democratic MC and Republican MC tweets, where greater diversity emerges with in-party owned issues, while conservatives tune in much more when Democrat MCs discuss Republican owned issues.


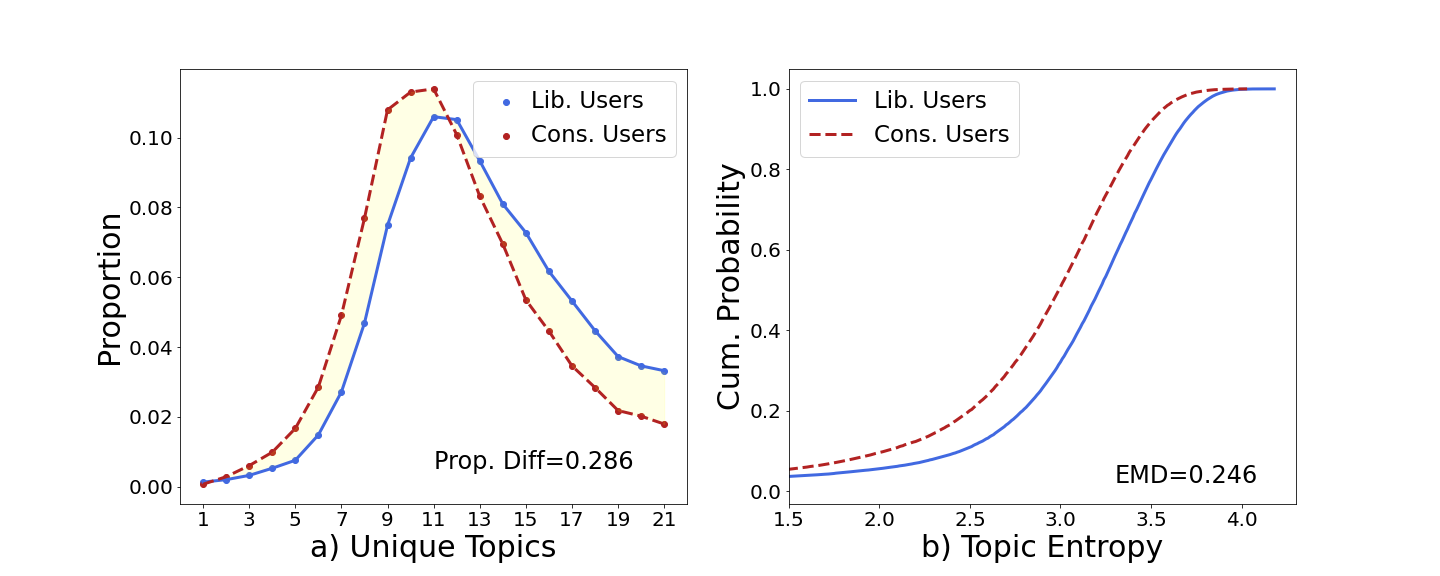


**Figure S8: Replication of Fig. 3 using DW Nominate scores, t**he proportional difference between liberals and conservatives with a) unique topics and b) entropy. The proportional difference increases to 0.286 from 0.194, whereas the EMD increase from 0.124 to 0.246. In other words, replication under DW nominate strengthens our findings.

**Table S1:** F1, recall, and precision per topic from deep learning model (using the BERTweet architecture). Each policy topic is labeled and derived from the comparative agenda project.

|  | **f1** | **recall** | **precision** | **validation count** |
| --- | --- | --- | --- | --- |
| Not Policy Related | 72% | 67% | 78% | 302 |
| Macroeconomics | 87% | 84% | 90% | 657 |
| Civil Rights | 90% | 88% | 92% | 403 |
| Health | 94% | 95% | 93% | 885 |
| Agriculture | 88% | 89% | 87% | 169 |
| Labor | 77% | 76% | 77% | 189 |
| Education | 91% | 93% | 89% | 281 |
| Environment | 78% | 78% | 77% | 165 |
| Energy | 85% | 83% | 87% | 193 |
| Immigration | 95% | 96% | 94% | 353 |
| Transportation | 85% | 89% | 82% | 122 |
| Law and Crime | 91% | 92% | 90% | 531 |
| Social Welfare | 85% | 89% | 83% | 96 |
| Housing | 86% | 92% | 80% | 53 |
| Domestic Commerce | 81% | 82% | 80% | 263 |
| Defense | 84% | 85% | 83% | 567 |
| Technology | 75% | 81% | 70% | 78 |
| Foreign Trade | 69% | 71% | 68% | 24 |
| International Affairs | 84% | 82% | 87% | 319 |
| Government Operations | 85% | 86% | 83% | 498 |
| Public Land | 67% | 68% | 65% | 109 |

**Precision** (also called Positive Predictive Value), measures how many of the predicted positive instances are actually positive; **Recall** (also called Sensitivity or True Positive Rate), measures how many of the actual positive instances are predicted correctly; **F1-Score** is the harmonic mean of precision and recall, providing a balance between the two.

**Table S2:** BERTweet classifier accuracy for policy versus not policy.

| **Label** | **Accuracy** |
| --- | --- |
| Policy | 0.926174 |
| Not Policy | 0.824503 |
| F1 Score | 0.88518 |

**Table S3:** 20 Comparative Agenda Project’s Policy topics and sensitivities to toxicity. Beta denotes the slope of toxicity versus retweets (virality)

| **Names** | **Democrat Beta** | **Republican Beta** | **Supply Diff.** |
| --- | --- | --- | --- |
| Defense | 1.95 | 1.24 | 17,578 |
| Macroeconomics | 3.13 | 0.03 | 9,148 |
| International Affairs | 0.55 | 0.30 | 5,246 |
| Energy | 2.49 | 0.92 | 3,931 |
| Agriculture | 2.33 | 0.64 | 1,647 |
| Domestic Commerce | 2.67 | 0.18 | 272 |
| Foreign Trade | 3.06 | 0.14 | 73 |
| Public Lands | 2.79 | 0.43 | 32 |
| Not Policy | 1.68 | 0.51 | -668 |
| Transportation | 2.40 | 0.95 | -1,552 |
| Housing | 3.10 | -0.39 | -1,796 |
| Technology | 3.07 | 0.49 | -2,347 |
| Social Welfare | 2.28 | 0.55 | -3,261 |
| Labor | 2.26 | 0.17 | -3,648 |
| Education | 2.73 | 0.82 | -5,869 |
| Government Operations | 2.86 | -0.40 | -6,775 |
| Immigration | 1.58 | 0.35 | -10,484 |
| Environment | 2.38 | 0.23 | -10,916 |
| Health | 1.65 | 0.07 | -14,168 |
| Law and Crime | 1.56 | -0.32 | -16,394 |
| Civil Rights | 1.25 | 0.01 | -16,749 |

**Table S4:** Policy versus non-toxic / toxic content, across Democrat and Republican MCs. The cut off for toxicity is the mean, which provides a looser cut-off for toxicity than the main results, which demonstrates robustness. Democrats engage policy with much more toxicity than Republicans (1.8 versus 1.3 non-toxic / toxic ratio). Furthermore, Democrats are in general more toxic (37% versus 29% of content).

| **(a) Democratic MCs** | Not Policy | Policy | **Not Policy + Policy** |
| --- | --- | --- | --- |
| Non-toxic | 0.263 | 0.365 | 0.628 |
| Toxic | 0.093 | 0.279 | 0.372 |
| **Non-toxic** **/ Toxic Ratio** | 2.815 | 1.310 |  |

| **(b) Republican MCs** | Not Policy | Policy | **Not Policy + Policy** |
| --- | --- | --- | --- |
| Non-toxic | 0.343 | 0.37 | 0.714 |
| Toxic | 0.102 | 0.184 | 0.286 |
| **Non-toxic / Toxic Ratio** | 3.35 | 2.01 |  |

**Table S5:** Test of Proportion counts for a) supply and b) demand.

|  | Toxic tweets | Total Tweets | Percentage Toxic |
| --- | --- | --- | --- |
| Democrats | 95,082 | 629,196 | 15.1% |
| Republicans | 63,546 | 648,149 | 9.8% |

|  | Toxic tweets | Total Retweets | Percentage Toxic |
| --- | --- | --- | --- |
| Liberals | 1,991,285 | 9,769,259 | 20.4% |
| Conservatives | 486,369 | 3,239,052 | 15.0% |

**Table S5: Summary statistics for the supply and demand**

| **Category** | **Statistic** | **Value** |
| --- | --- | --- |
| Supply Dataset | Unique MCs | 831 |
|  | Mean Incivility | 0.135 |
|  | Mean retweets | 111.6 |
|  | Median retweets | 4 |
| Demand Dataset | Total retweet events | 13,581,100 |
|  | Unique Retweeting Users | 1,591,290 |
|  | Average political score | -0.554 |
|  | Median political score | -0.76 |
|  | Average nominate score | -0.026 |
|  | Median nominate score | -0.26 |
|  | Mean retweets | 8.26 |
|  | Median retweets | 1 |

**Table S6: Regression results with residual check for Figures 4a and 4b**

| **Regression Subset** | **Incivility Coefficient** | **Durbin-Watson (residual autocorr.)** | **P-value** |
| --- | --- | --- | --- |
| Democrat in-group | 0.4136 | 1.439 | 2.54E-22 |
| Democrat out-group | 0.7585 | 1.543 | 7.28E-220 |
| Republican in-group | 0.5469 | 1.462 | 2.59E-40 |
| Republican out-group | 0.8929 | 1.494 | 7.84E-167 |
| Republican in-group | 2.3976 | 1.984 | 0.00E+00 |
| Republican out-group | 1.5337 | 1.995 | 0.00E+00 |
| Republican in-group | 0.7799 | 1.988 | 0.00E+00 |
| Republican out-group | -0.0944 | 1.985 | 1.03E-09 |

**Table S7: CatBoost regressor training and error terms**

| **Parameter** | **Republican Model** | **Democrat Model** |
| --- | --- | --- |
| Iterations | 500 | 500 |
| Learning Rate | 0.2 | 0.2 |
| Depth | 8 | 8 |
| L2_leaf_reg | 0.2 | 0.2 |
| RMSE | 0.6 | 0.71 |
| R² | 0.11 | 0.17 |
